# Segmental Assessment of Trunk Control in infants from 4 to 9 months of age- a psychometric study

**DOI:** 10.1186/s12887-018-1153-4

**Published:** 2018-05-31

**Authors:** Tamis W. Pin, Penelope B. Butler, Hon-Ming Cheung, Sandra Lai-Fong Shum

**Affiliations:** 1 Department of Rehabilitation Sciences, The Hong Kong Polytechnic University, Hung Hom, Kowloon, Hong Kong; 2 Health, Exercise and Active Living, Manchester Metropolitan University, Manchester, UK; 3 Department of Paediatrics, Prince of Wales Hospital, Shatin, Hong Kong; 4 Physiotherapy Department, Prince of Wales Hospital, Shatin, Hong Kong

**Keywords:** Postural balance, Trunk control, Reliability, Validity, Responsiveness, Psychometric, Infants

## Abstract

**Background:**

Efficient trunk control is crucial in infant motor development when infants first learn how to move against gravity. Traditional assessments of trunk control commonly treat the trunk as one unit but the Segmental Assessment of Trunk Control (SATCo) assesses trunk control segment by segment. Good reliability and validity of the SATCo have been proved in children with neuro-disability but not yet validated in young infants. The present study was to examine if the SATCo was reliable, valid and responsive for infants aged 4 to 9 months.

**Methods:**

Infants born at full-term and at less than 30 weeks of gestation were recruited and assessed using the SATCo monthly from 4 to 9 months of age (corrected for prematurity). Intra-class correlation coefficients (ICC) were used to examine intra- and inter-rater reliability between 2 raters. The ability of the SATCo to demonstrate differences between the full-term and preterm infants was examined using the Mann Whitney U test. The responsiveness of the SATCo on the full-term infants was tested using the Friedman test.

**Results:**

Twenty full-term (mean gestation = 38.7 weeks; birthweight = 3019.9 g) and 20 preterm infants (mean gestation = 27.2 weeks; birthweight = 989.6 g) were recruited. The intra and inter-rater reliability of the SATCo levels on full-term infants was good (all ICC > 0.75), except inter-rater reliability at 6 months. The preterm infants scored significantly lower in reactive trunk control at 8 months (Mann Whitney U = 102.0, *p* = 0.016) but this was the only difference noted. A significant developmental trend was shown in the static, active and reactive trunk control of the full-term infants (Chi-square = 81.4, 75.6 and 79.5 respectively, all *p* < 0.001.

**Conclusions:**

The SATCo was reliable and responsive in assessing trunk control in young infants aged from 4 to 9 months. Care should be exercised when testing infants aged 5 to 6 months, who are more likely to use subtle hand support, and for those who have already achieved independent sitting. The SATCo could differentiate the reactive trunk control between the full-term and preterm infants at 8 months but not earlier. Psychometric properties of the SATCo in infants with motor disorders requires further investigation.

## Background

Efficient postural control of the trunk (trunk control) allows an individual to perform a variety of tasks in an upright, vertical posture without loss of balance and plays a significant role in motor development as an infant learns to move against gravity [1, 2]. Trunk control emerges in the first 12 months after birth for typically developing infants but is commonly delayed in children with motor impairments [1].

Assessment of trunk control in infants and young children can include kinematic and kinetic measures [2], but these are generally limited to research laboratories and are not clinically practical. Thus, a developmental assessment in the clinic usually incorporates an assessment of trunk control [1]. However, the main limitation of these developmental assessments is that the trunk is considered as a single unit, without consideration of the different trunk segments [3]. Thus differentiation cannot be made to determine the relationship between upright segmental trunk control and achievement of the major motor milestones. As an example, does an infant need to have gained assured control when upright at the lower thoracic, upper or lower lumbar segment in order to crawl on all-fours or to sit independently? The conventional developmental assessment thus neither correlates development of trunk control with overall motor development nor, importantly for infants or children with motor impairments, how development of neutral vertical control in specific trunk segments could contribute to better motor function. An atypical motor developmental profile has been identified in preterm infants; imbalance of flexor and extensor muscle strength has been postulated as contributing to poor trunk control in upright positions in the first 18 months of corrected age [4–6]. However, the contribution of any segmental influence to this profile is unknown.

The Segmental Assessment of Trunk Control (SATCo) provides in-depth segment by segment assessment of trunk control [3]. The child’s trunk control is examined by progressively reducing the support from the shoulder girdle to assess head control, through support at the axillae (upper thoracic), inferior scapula (mid-thoracic), lower ribs (lower thoracic), below ribs (upper lumbar), pelvis (lower lumbar) and no support to assess full trunk control [3]. Trunk control is tested under 3 different conditions in sitting: maintenance of a neutral vertical posture with no movement (static control), maintenance of the neutral vertical posture during voluntary head or reaching movements (active control) and recovery of the neutral posture after a disturbance of balance by a nudge (reactive control) [3]. The preliminary results of the SATCo showed a high inter-rater reliability (Intra-class correlation, ICC ≥ 0.8) [3, 7] and moderate to good correlations with other established motor assessments (r from 0.65 to 0.88) [3]. Nevertheless, in the psychometric study of the SATCo by Butler and colleagues [3], just eight typically developing infants (aged 3 to 9 months) and one infant with neurodevelopment delay (aged 18-months) were tested. A very small number out of 31 children with cerebral palsy was recruited in the other SATCo psychometric study [7]. Although the SATCo is a promising outcome measure of segmental trunk control, its psychometric properties in infants and young children should be fully examined since psychometric evaluation of an outcome measure is population-specific [8]. As there is no previous study focussing solely on the psychometric properties of the SATCo in young infants, it would be reasonable to evaluate the psychometric characteristics in typically developing young infants before testing on infants with neurodisability.

The present study focused on determination of the reliability, construct validity and responsiveness of the SATCo in young infants. According to the consensus statement from the COSMIN initiative (COnsensus-based Standards for the selection of health Measurement INstruments) [9], reliability is defined as the consistency of the measurement from the outcome measure in the absence of real changes among the assessors (both intra- and inter-rater reliability) [9]. Construct validity represents whether the outcome measure is able to demonstrate differences between relevant groups [9]. Responsiveness refers to the ability of the outcome measure to detect changes over time [9]. This study was to investigate whether the SATCo:

1. was reliable in assessing trunk control in typically developing full-term (FT) infants from 4 to 9 months of age;
2. could differentiate between preterm (PT) and FT born infants aged from 4 to 9 months (all ages were corrected for prematurity in the remaining text); and
3. could demonstrate changes over time in the FT infants from 4 to 9 months of age.

## Methods

### Participants

The FT infants were recruited via personal contact or by word of mouth. The inclusion criteria were infants born at or after 37 weeks of gestation with no concerns expressed by parents or family doctor about their prenatal and perinatal histories, or postnatal development. The PT infants were recruited from a neonatal intensive care unit in one of the seven cluster hospitals in Hong Kong and via a private internet parental group whose infants were all born prematurely. PT infants were included if born at or less than 30 weeks of gestation. Both PT and FT infants with known congenital abnormalities and syndromes, such as Down syndrome, were excluded. As co-morbidities from prematurity, such as chronic lung disease, intra-ventricular haemorrhage, necrotising enterocolitis, and retinopathy of prematurity are common, infants with these co-morbidities were not excluded but this information was noted for any future analyses. This research study was conducted in accordance with the Declaration of Helsinki. Ethics approvals were granted from the Departmental Research Committee, Department of Rehabilitation Sciences, The Hong Kong Polytechnic University (HSEARS20140214001) and Joint Chinese University of Hong Kong- New Territories East Cluster Clinical Research Ethics Committee (2014.193). Parents of all participating infants signed an informed consent prior to the assessment.

### Measures and procedure

All FT and PT infants were longitudinally followed up from 4 to 9 months old and assessed using the SATCo monthly by the first author (TWP) at the infants’ homes. A monthly visit was considered appropriate to capture the rapid gross motor development in young infants without putting an excessive burden on the study families with the home visits. The SATCo testing was conducted according to the published criteria, although the infants were allowed to wear thin unrestrictive clothing instead of being trunk naked during testing [3]. The infants’ trunk control was scored as ‘present’, ‘absent’ or ‘not tested’ at each trunk segmental level under each of the three conditions (static, active and reactive). The infants’ performance was videotaped with two cameras set on tripods at 45° and 90° to the infant respectively.

A sample size of 11 infants was required to achieve 80% power and α = 0.15 with 2 observations per subject and an effect size of 0.2 (H_o_ = 0.7 and H_1_ = 0.9) [10]. Twenty FT infants were targeted for this study and 20 PT infants also targeted for comparison in the validity component.

### Data analyses

In order to statistically analyse the SATCo levels, a number was assigned for each trunk segmental level where control was being learnt: 1 for head control, 2 for upper thoracic level, 3 for mid-thoracic, 4 for lower thoracic, 5 for upper lumbar, 6 for lower lumber and 7 for full trunk control. The number 8 was used if full trunk control was demonstrated; this was the same process as in the previous psychometric study of the SATCo [3]. Each infant thus had 3 numerical values indicating their respective segmental levels of learning static, active and reactive control for each month. For example, if the infant had 5, 4 and 3, this implied that static trunk control was being learned at upper lumbar level, active trunk control at lower thoracic and reactive trunk control at mid thoracic.

To examine the intra- and inter-rater reliability of the SATCo, the first (TWP) and second (PBB) authors independently graded the FT infants. PBB is the author of the SATCo [3] and TWP is a paediatric physiotherapist with over 25 years of clinical experience. TWP had a one-week training with PBB prior to the present study to establish consensus. The video-recordings were anonymously coded with no indication of the age and name of the infant to reduce the bias of scorings by PBB. This method has been previously validated by a psychometric study of the SATCo [7]. For the intra-rater reliability, 10% of the video-recordings was randomly drawn from a hat by a research assistant, who also ensured that no 2 video-recordings came from the same infant. These video-recordings were independently re-scored by the same two authors without any knowledge of previous SATCo scores given. The two scorings took place at least 4 weeks apart to minimise recall bias. In order to take rank orders of measurements of the study infants into consideration, more rigorous intra-class correlation coefficients (ICC) was used to analyse the intra- and inter-rater reliability, despite the fact that the SATCo is ordinal data [11]. The sum of the 3 numerical values from the SATCo of each infant was used to calculate the ICC of inter-rater reliability. Intra-class correlation coefficients were used to examine intra- and inter-rater reliability. If the coefficient is less than 0.5, it is considered to have poor reliability; the coefficient between 0.51 and 0.75 represents moderate reliability; and the coefficient greater than 0.75 represents good reliability [11].

To examine if the SATCo could differentiate the PT infants from their FT peers, the three SATCo scores of the PT and FT infants from 4 to 9 months old were compared using the Mann Whitney U test. The SATCo levels of the PT and FT infants from the first author (TWP) were used. The Friedman test was used to examine the responsiveness of the SATCo on the FT infants, i.e. changes over time [11]. All statistical significance levels were set at *p* ≤ 0.05. No adjustment to the significance level was made.

## Results

Twenty FT infants (mean gestation = 38.7 weeks, SD 1.0; mean birthweight = 3019.9 g, SD 370.7; 60% males) were recruited and assessed between June 2014 and May 2015. Twenty PT infants were recruited and assessed from December 2014 to July 2016 (mean gestatio*n* = 27.2 weeks, SD 1.7; mean birthweight = 989.6 g, SD 237.0; 45% males) (Appendix). Two data points were missing (1.6%) in the FT group (*n* = 1 sick infant and *n* = 1 family away) and 9 (7.5%) in the PT group (*n* = 4 late start of data collection, *n* = 2 family away, *n* = 2 unable to be tested due to constant crying and struggling during the test, and *n* = 1 sick infant). As expected, there were significant differences in gestation age (*t* = − 25.5, *p* < 0.001) and birthweight (*t* = − 20.6, *p* < 0.001) between the two groups but no difference was found in gender (Chi square = 0.90, *p* = 0.342) or number of infants with birthweight appropriate for gestation (Chi Square = 0.36; *p =* 0.500).

### Reliability

Both examiners (TWP and PBB) independently scored 118 video-recordings of the FT infants (*n* = 20 for each month, but only 18 at 9 months of age). Both examiners re-scored 17 of these 118 video-recordings (*n* = 2 for 4 months; *n* = 3 for 5 and 6 months; *n* = 4 for 7 months; *n* = 3 for 8 months; *n* = 2 for 9 months). Table 1 shows the intra- and inter-rater reliability of the SATCo scores of the FT infants. The intra-rater reliability for both examiners was good with the ICC consistently well above 0.75 with all *p* < 0.001 (Table 1). The inter-rater reliability was also good with the ICC above 0.75, except at 6 months when the ICC was 0.641, with all *p* ≤ 0.015 (Table 1).

**Table 1:** Intra- and Inter-rater reliability of SATCo

	ICC	95% CI
Intra-rater reliability (*n* = 17)		
Examiner 1 (first author, TWP)		
Static control	0.987	0.964, 0.995
Active control	0.982	0.949, 0.993
Reactive control	0.985	0.985, 0.994
Examiner 2 (second author, PBB)		
Static control	0.976	0.935, 0.991
Active control	0.978	0.938, 0.992
Reactive control	0.964	0.900, 0.987
Inter-rater reliability (2 examiners on 1 occasion)		
4 months (*n* = 20)	0.895	0.736, 0.959
5 months (*n* = 20)	0.761	0.397, 0.905
6 months (*n* = 20)	0.641	0.094, 0.858
7 months (*n* = 20)	0.967	0.917, 0.987
8 months (*n* = 20)	0.812	0.524, 0.925
9 months (*n* = 18)	0.873	0.660, 0.952

All ICC significant at *p ≤* 0.015 at two-way mixed effects model on average measures with absolute consistency definition

*n* number of infants at each month

### Construct validity

No significant difference was found between the PT and FT groups in the SATCo segmental levels from 4 to 9 months (all *p* > 0.05), except for reactive trunk control at 8 months (Mann Whitney U = 102.0, *p* = 0.016) (Table 2). A borderline significant difference was found in static trunk control at 8 months (Mann Whitney U = 121.5, *p* = 0.058) and in reactive trunk control at 9 months (Mann Whitney U = 111.5, *p* = 0.057).

**Table 2:** Comparison of the median SATCo segmental levels between the preterm and full-term born infants

		Preterm^a^	Full-term^a^
SATCo at 4 m *N* = 17 preterm *N* = 20 full-term	Static	5 (1–5)	5 (4–5)
	*p*	0.670	
	Active	4 (1–5)	4.5 (3–5)
	*p*	0.308	
	Reactive	4 (1–5)	4 (3–5)
	*p*	0.220	
SATCo at 5 m *N* = 19 preterm *N* = 20 full-term	Static	5 (4–6)	5 (4–6)
	*p*	0.075	
	Active	5 (3–6)	5 (4–6)
	*p*	0.629	
	Reactive	5 (3–5)	5 (4–6)
	*p*	0.304	
SATCo at 6 m *N* = 18 preterm *N* = 20 full-term	Static	6 (4–7)	6 (4–7)
	*p*	0.403	
	Active	6 (5–6)	6 (4–7)
	*p*	0.856	
	Reactive	5 (4–6)	5 (4–6)
	*p*	0.509	
SATCo at 7 m *N* = 20 preterm *N* = 20 full-term	Static	6 (5–8)	6.5 (5–8)
	*p*	0.467	
	Active	6 (4–7)	6 (5–8)
	*p*	0.126	
	Reactive	6 (4–7)	6 (5–7)
	*p*	0.305	
SATCo at 8 m *N* = 18 preterm *N* = 20 full-term	Static	7 (5–8)	8 (6–8)
	*p*	0.058	
	Active	6.5 (5–8)	7.5 (5–8)
	*p*	0.082	
	Reactive	5.5 (5–8)	7 (6–8)
	*p*	0.016	
SATCo at 9 m *N* = 19 preterm *N* = 18 full-term	Static	8 (4–8)	8 (7–8)
	*p*	0.091	
	Active	8 (4–8)	8 (7–8)
	*p*	0.184	
	Reactive	7 (3–8)	7 (6–8)
	*p*	0.057	

Ages corrected for the preterm born infants. Numbers in brackets = range of SATCo scores

^a^The numbers represent medians of the SATCo trunk segmental level at which control was being learnt: 1 = head control, 2 = upper thoracic level, 3 = mid-thoracic, 4 = lower thoracic, 5 = upper lumbar, 6 = lower lumber, 7 = full trunk control, and 8 = full trunk control achieved. Please note that the SATCo is an ordinal scale and the learning levels shown in this table were the medians of each group of infants at each month. The non-integral numbers reported in the table were purely for statistical purposes. In real life situations, no half-level would be credited to the infants

**Fig. 1:**
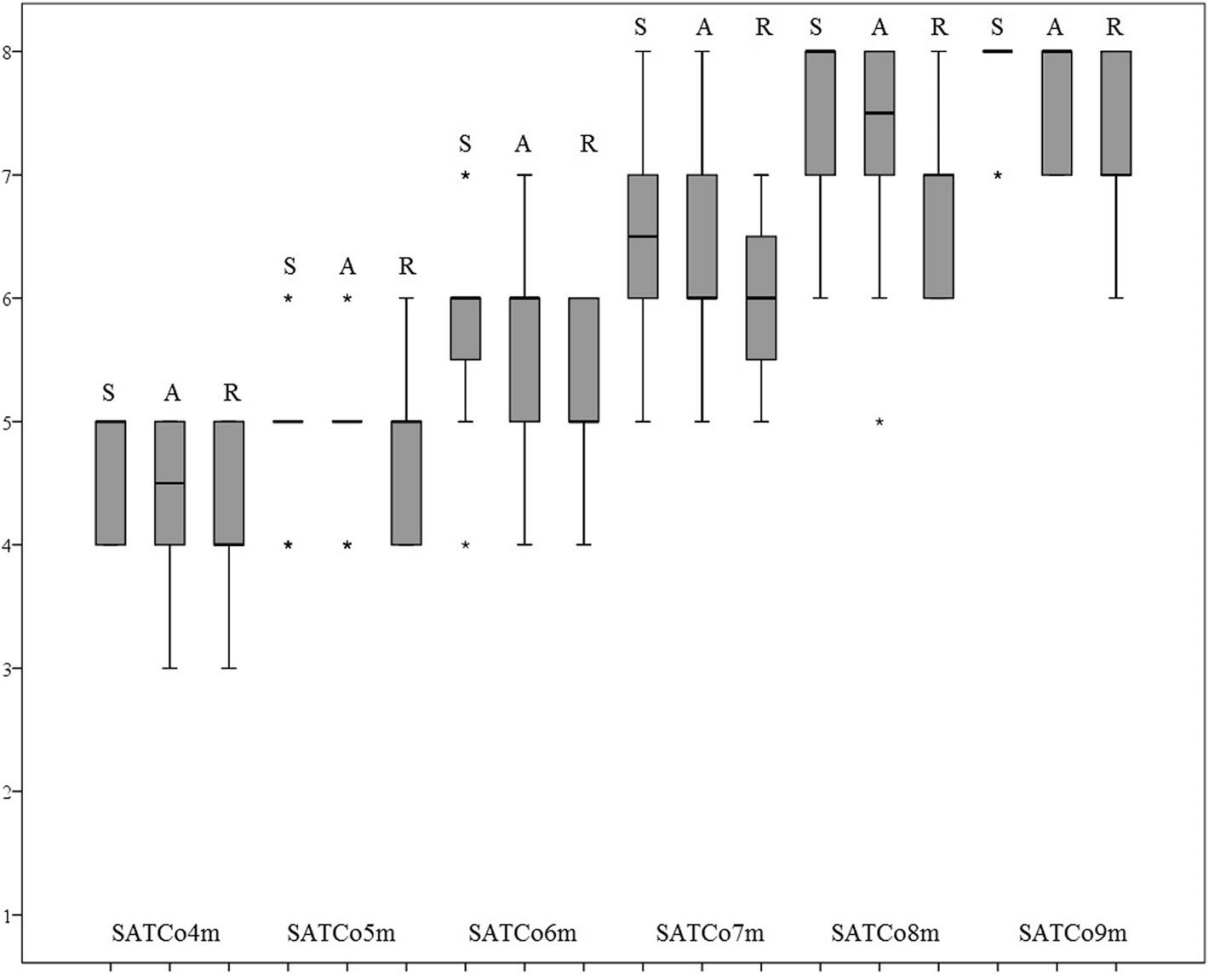
Developmental trend of trunk control from 4 to 9 months of age in full-term infants. S- static control, A- active control, R- reactive control. Numbers on the y-axis are the SATCo trunk segmental level at which control was being learnt (1 = head control, 2 = upper thoracic level, 3 = mid-thoracic, 4 = lower thoracic, 5 = upper lumbar, 6 = lower lumber, 7 = full trunk control, and 8 = full trunk control achieved). The solid line represents the medians of the group at each age group. The boxes and the whiskers represent the spread of the data within that age group. The asterisks represent outliers in that age group

For the responsiveness of the SATCo, a significant time effect was shown in the static, active and reactive segmental trunk control of the FT infants (Chi-square = 81.4, 75.6 and 79.5 respectively, all *p* < 0.001) (Fig. 1). Paired comparisons at each month, i.e. 4 with 5 months, 5 with 6 months, and so on, showed a significant time effect in the static, active and reactive trunk control of the infants at all ages (all *p* < 0.05), with the exception of a borderline significance in the active control between 4 and 5 months (Chi-square = 3.6, *p* = 0.058) and reactive control between 8 and 9 months (Chi-square = 3.6, *p* = 0.058).

### Availability of data and materials

The raw data and data sets used and analyzed in the present study are available from the corresponding author on reasonable request.

## Discussion

The SATCo emphasises a segmental assessment of trunk control, making it unique among other developmental assessments and assessments of trunk control [3]. The present study has expanded on previous work [3, 7] by specifically examining its reliability, construct validity and responsiveness in young infants aged from 4 to 9 months.

### Reliability

For FT infants aged from 4 to 9 months, the inter-rater reliability of the SATCo was good from 4 to 9 months, with an exception at 6 months which showed only fair reliability (Table 1). The intra-rater reliability was shown to be good (Table 1). These results are comparable with the previously reported reliability [3, 7]. Although the ICC for the inter-rater reliability was in general over 0.75, except at 6 months, the confidence intervals (CI) were relatively wide at 5, 8 and 9 months. Most of the FT infants aged 8 to 9 months were able to sit independently without any hand support and achieved full trunk control under the three conditions in the SATCo (Table 2). These infants were able to move off the testing bench if not restrained. It was challenging to keep these active infants on the testing bench with the correct starting position for sufficient time to test static, active and reactive full trunk control. The failure to recognise subtle hand support by the child, such as one hand resting on the lap especially during the test for reactive control, was the most common problem, and was identified as a common testing error in the previous psychometric study of the SATCo [3]. These factors may have contributed to the wide CI at 5, 8 and 9 months and the lower inter-rater reliability at 6 months. However, despite the wide CI, over 75% of the results between the 2 examiners were in total agreement and discrepancy of more than 2 segmental levels between the 2 examiners was only 6.7% in total. These findings are in agreement with previous findings on school-aged children with cerebral palsy [7].

These results suggest that the SATCo is appropriate for typically developing infants aged 4 to 9 months of age, with care particularly with respect to hand support with infants aged at 5 and 6 months and for those infants who have already achieved independent sitting. It could be helpful for an extra assistant, if available, to watch vigilantly for subtle hand support or other compensations that the child might use during the test to potentially improve the reliability of the SATCo. If possible, training in the SATCo with more experienced users could help to avoid these pitfalls.

### Construct validity

The SATCo was able to differentiate PT from FT infants in their reactive trunk control at 8 months of age but not at other ages. It has been postulated that an imbalance in the development of flexor and extensor muscle strength in PT infants may adversely impact their trunk control [4–6]. These previous studies demonstrated the significant differences between the PT and FT infants by assessing their gross motor skills in various positions [4–6]. The present study specifically assessed the segmental trunk control in an upright position. All infants aged 4 to 6 months, regardless FT or PT, spend the majority of their time in reclined positions and thus the demand on their vertical trunk control would be similar for both groups of infants (Table 2). This was reflected by the lack of a significant difference in the SATCo scores at this young age range. From 7 months onwards, most of the FT infants were fully mobile on the floor and were able to sit independently. The demand of their trunk control would thus be very different from the PT infants, who were still at the stage of mastering independent sitting and floor ambulation. This has been reflected in the significant difference in reactive trunk control at 8 months between the two groups of infants. The borderline significant difference in active trunk control at 8 months may be due to the small sample size of the present study.

It is also possible that the majority of the PT infants in the present study were neurologically intact infants (Appendix); any subtle difficulties with trunk control might only become apparent at an older age with increasing demand on their trunk control in a vertical posture such as standing [4, 6]. A larger sample size of infants for a longer follow-up duration may verify this speculation. At present, it is reasonable to conclude that the SATCo is able to differentiate the PT and FT infants at 8 months of (corrected) age but not earlier in their reactive trunk control. The construct validity of the SATCo for infants against another valid outcome measure, such as developmental assessments, requires further investigation before its full clinical use for young infants.

### Responsiveness

The results of the Friedman test showed that the SATCo was responsive in demonstrating the changes in static, active and reactive segmental trunk control in the FT infants over time from 4 to 9 months of age (Fig. 1). High or maximal SATCo scores were demonstrated at 8 to 9 months and contrasted clearly with the scores at 4 to 5 months of age for the infants (Fig. 1). Furthermore, it appears that neutral vertical trunk control develops cephalo-caudally and, among the three types of trunk control, reactive control appears to be the last to become established in infants of this age range (Fig. 1).

### Study limitations

This study consisted of a relatively small sample size of both FT and PT infants, despite the fact that sample size was calculated a priori. Caution should be taken when interpreting the present results, especially for some of the reliability data with a wider CI.

## Conclusions

The SATCo has been shown to be a generally reliable outcome measure for examining segmental trunk control of young infants aged from 4 to 9 months. Wide CIs were found at 5, 8 and 9 months of age and reduced reliability at 6 months. Thus, close attention should be taken during testing of infants who have already achieved independent sitting and are more likely to fidget and move and at ages where an infant may use subtle hand support, for example around 5 to 6 months. The SATCo was able to differentiate between PT and FT infants in their reactive trunk control at 8 months of (corrected) age but not earlier as would be anticipated from the reclined postures used by all infants at a younger age. In the FT infants, trunk control was shown to develop in a cephalo-caudal direction and to become fully established around 8 to 9 months of age: reactive trunk control emerged after static and active control at each monthly SATCo test. Further investigations of the psychometric properties of the SATCo in infants with neuro-disability is recommended before it is widely used in clinical and research settings.

## Appendix

**Table 3:** Characteristics of the preterm infants

	GA	BW	Gender	AGA^b^	IVH	ROP	CLD	NEC
PT001	26.6	1070	M	Yes				Yes
PT003	29	1300	M	Yes				
PT004	28.1	1310	F	Yes				
PT005	26.7	908	M	Yes	Yes (left grade 2)		Yes	
PT006	26	890	M	Yes	Yes (left grade 2)		Yes	
PT007	27.4	940	F	Yes	Yes (left grade 1)		Yes	
PT008	27.6	675	M	No	Yes (left grade 2)	Yes	Yes	
PT009	25	790	F	Yes	0		Yes	
PT011	28.9	1225	M	Yes	0			
PT012	26.1	800	F	Yes	0		Yes	
PT013	26.3	780	F	Yes	Yes (right grade 2)		Yes	
PT014	25.6	745	M	Yes		Yes	Yes	
PT015	26.6	950	F	Yes			Yes	
PT016^a^	28.6	1080	M	Yes	Yes (right grade 4, left grade 3)		Yes	
PT017	26.9	1000	F	Yes	0	Yes	Yes	
PT018	23.6	558	F	Yes	0	Yes	Yes	
PT019	30	1220	F	Yes	Yes (left grade 1)		Yes	Yes
PT020	30	1210	F	Yes			Yes	
PT021	29.6	1440	F	Yes				
PT022	26	900	M	Yes			Yes	

*AGA* weight appropriate for gestation age, *BW* birthweight, *CLD* chronic lung disease, *CP* cerebral palsy, *GA* gestation age, *IVH* intra-ventricular haemorrhage, *NEC* necrotising enterocolitis, *ROP* retinopathy of prematurity

^a^This infant was diagnosed with cerebral palsy at 8 months corrected age

^b^Fok TF, So HK, Wong E, Ng PC, Chang A, Lau J, et al. Updated gestations age specific birth weight, crown-heel length, and head circumference of Chinese newborns. Arch Dis Child Fetal Neonatal Ed. 2003;88:F229–36

## Abbreviations

FT: Full-term
PT: Preterm
SATCo: Segmental Assessment of Trunk Control
